# Short-Lived Immunity After 17DD Yellow Fever Single Dose Indicates That Booster Vaccination May Be Required to Guarantee Protective Immunity in Children

**DOI:** 10.3389/fimmu.2019.02192

**Published:** 2019-09-26

**Authors:** Ana Carolina Campi-Azevedo, Laise Rodrigues Reis, Vanessa Peruhype-Magalhães, Jordana Grazziela Coelho-dos-Reis, Lis Ribeiro Antonelli, Cristina Toscano Fonseca, Christiane Costa-Pereira, Elaine Maria Souza-Fagundes, Ismael Artur da Costa-Rocha, Juliana Vaz de Melo Mambrini, Jandira Aparecida Campos Lemos, José Geraldo Leite Ribeiro, Iramaya Rodrigues Caldas, Luiz Antônio Bastos Camacho, Maria de Lourdes de Sousa Maia, Tatiana Guimarães de Noronha, Sheila Maria Barbosa de Lima, Marisol Simões, Marcos da Silva Freire, Reinaldo de Menezes Martins, Akira Homma, Pedro Luiz Tauil, Pedro Fernando Costa Vasconcelos, Alessandro Pecego Martins Romano, Carla Magda Domingues, Andréa Teixeira-Carvalho, Olindo Assis Martins-Filho

**Affiliations:** ^1^Instituto René Rachou, Fundação Oswaldo Cruz – FIOCRUZ-Minas, Belo Horizonte, Brazil; ^2^Departamento de Fisiologia e Biofísica, Universidade Federal de Minas Gerais, Belo Horizonte, Brazil; ^3^Secretaria Municipal de Saúde de Belo Horizonte, Belo Horizonte, Brazil; ^4^Secretaria do Estado de Saúde de Minas Gerais, Belo Horizonte, Brazil; ^5^Escola Nacional de Saúde Pública – FIOCRUZ, Rio de Janeiro, Brazil; ^6^Instituto de Tecnologia em Imunobiológicos Bio-Manguinhos – FIOCRUZ, Rio de Janeiro, Brazil; ^7^Faculdade de Medicina, Universidade de Brasília, Brasilia, Brazil; ^8^Instituto Evandro Chagas, Ananindeua, Brazil; ^9^Departamento de Imunização e Doenças Transmissíveis (DEIDT) – Secretaria de Vigilância em Saúde, Ministério da Saúde, Brasilia, Brazil; ^10^Programa Nacional de Imunizações – Secretaria de Vigilância em Saúde, Ministério da Saúde, Brasilia, Brazil

**Keywords:** yellow fever, 17DD vaccine, children, neutralizing antibodies, cellular memory

## Abstract

The Yellow Fever (YF) vaccination is recommended for people living in endemic areas and represents the most effective strategy to reduce the risk of infection. Previous studies have warned that booster regimens should be considered to guarantee the long-term persistence of 17DD-YF-specific memory components in adults living in areas with YF-virus circulation. Considering the lower seroconversion rates observed in children (9–12 months of age) as compared to adults, this study was designed in order to access the duration of immunity in single-dose vaccinated children in a 10-years cross-sectional time-span. The levels of neutralizing antibodies (PRNT) and the phenotypic/functional memory status of T and B-cells were measured at a baseline, 30–45 days, 1, 2, 4, 7, and 10 years following primary vaccination. The results revealed that a single dose induced 85% of seropositivity at 30–45 days and a progressive time-dependent decrease was observed as early as 2 years and declines toward critical values (below 60%) at time-spans of ≥4-years. Moreover, short-lived YF-specific cellular immunity, mediated by memory T and B-cells was also observed after 4-years. Predicted probability and resultant memory analysis emphasize that correlates of protection (PRNT; effector memory CD8^+^ T-cells; non-classical memory B-cells) wane to critical values within ≥4-years after primary vaccination. Together, these results clearly demonstrate the decline of 17DD-YF-specific memory response along time in children primarily vaccinated at 9–12 months of age and support the need of booster regimen to guarantee the long-term persistence of memory components for children living in areas with high risk of YF transmission.

## Introduction

The Yellow fever (YF) is an acute viral hemorrhagic disease caused by a single-stranded RNA Flavivirus that is endemic in Africa, South America and Central America (1, 2). YF is considered to be a re-emerging public health problem due to increasing number of outbreaks reported in the recent years worldwide (3).

The live attenuated YF vaccine has been an effective and safe control measure available to prevent YF since the 1930s (4). The YF vaccination is recommended for travelers and residents of endemic areas as the most effective strategy to reduce the risk of infection (5). The maintenance of high levels of immunity to YF is necessary to prevent the spread of the disease and large scale access to YF vaccines is critical to establish and maintain high levels of immunity amongst adult and children (3).

According to the World Health Organization, a single dose of YF vaccine is sufficient to provide lifelong protection in the general population (5, 6). However, previous studies have warned that the levels of neutralizing antibodies and the cellular immune responses elicited by YF vaccination decline considerably after primary vaccination (7–14).

Considering that the seroconversion rates observed in children following primary vaccination at 9–12 month of age are already lower than those observed in adults (15), it is expected that the duration of immunity in children would be even shorter as compared to adults. The present study was designed to assess the duration of humoral and cellular immunity following a single dose of 17DD-YF vaccine in children in a 10-year cross-sectional time-span. The quantification of neutralizing antibodies titers (PRNT) and the assessment of phenotypic/functional status of cellular memory were measured at baseline, 30–45 days, 1, 2, 4, 7, and 10 years following primary vaccination. These parameters have been considered relevant proxies of protection and can allow the monitoring of YF-specific immunological memory induced by the 17DD-YF vaccine (16–18).

This study aims to cover the gap in information about the duration of neutralizing antibodies and 17DD-specific T and B-cell memory overtime following the primary vaccination regimen in children. The data presented here bring original insights to support the importance of 17DD-YF booster vaccination in children to restore the YF-specific immune response elicited by primary vaccination.

## Methods

### Study Oversight

This study was sponsored by the Programa Nacional de Imunizações-PNI, Ministry of Health, Brazil. The protocol was approved by the research ethics committee at the Escola Nacional de Saúde Pública (CAAE 0014.0.031.000-10, February 20th 2010) as well as at Instituto René Rachou (CAAE 0023.0.245.000-10, February 11th 2011 and CAAE 25315213.6.0000.5091, May 23rd 2015) and registered at the Clinicaltrials.gov (NCT 02990182, January 9th 2015). Written informed consent was obtained from the parents of the participants in this study.

### Study Participants and Design

The study population consisted of 673 healthy children, from both genders, with ages ranging from 9 months to 12 years. Participants resided in two municipalities: Contagem and Ribeirão das Neves at Minas Gerais State, Brazil, and these two municipalities had no reports of YF cases for several decades prior the study onset. Moreover, the surveillance for epizootic events had not detected the circulation of YF virus amongst non-human primates in the State of Minas Gerais at the time of the study development. All participants have received, at 9–12 months of age, a single dose of the 17DD-YF substrain vaccine, produced by Instituto de Tecnologia em Imunobiológicos Bio-Manguinhos (FIOCRUZ, Brazil), from the seed lot 993FB013Z. The study was designed and supervised by the authors and structured into “two non-concurrent arms”: (i) the first arm was a paired longitudinal analysis to identify early correlates of protection and included two groups, referred as “NV(day 0)”—non-vaccinated children at baseline, *n* = 47 and “PV(day 30–45)”—vaccinees at 30–45 days after primary vaccination, *n* = 47; (ii) the second arm was a cross-sectional analysis comprising of five groups, categorized according to the time after 17DD-YF primary vaccination: “PV(year 1)”—vaccinees at 1 year (8–18 months) after primary vaccination, *n* = 141; “PV(year 2)”—vaccinees at 2 years (19–30 months) after primary vaccination, *n* = 114; “PV(year 4)”—vaccinees at 4 years (31–69 months) after primary vaccination, *n* = 128; “PV(year 7)”—vaccinees at 7 years (75–99 months) after primary vaccination, *n* = 116 and “PV(year 10)”—vaccinees at 10 years (101–142 months) after primary vaccination, *n* = 127.

Heparinized blood samples (7 mL) were collected at health units at Contagem and Ribeirão das Neves (MG, Brazil) and transported to Grupo Integrado de Pesquisas em Biomarcadores at Instituto René Rachou-FIOCRUZ-Minas in Belo Horizonte (MG, Brazil). Blood samples were centrifuged to obtain the plasma that was aliquoted into cryovials and stored at −80°C for further analysis of neutralizing antibodies against yellow fever virus by plaque-reduction neutralization test (PRNT). Mononuclear cells were also isolated to quantify the levels of 17DD-YF specific cellular memory response by *in vitro* phenotypic and functional analyses. In addition to blood collection, a questionnaire was applied to obtain demographic data. The vaccination status and the date of YF immunization was verified in the vaccination card. Current health status, the use of prescribed medicine, pathological conditions, and any travel history after 17DD-YF vaccination was registered. Eligibility criteria included children of both genders with primary 17DD-YF vaccination at 9–12 months of age, with post-vaccination time ranging from 30–45 days to 10 years. Exclusion criteria encompassed the presence of autoimmune diseases, hemoglobinopathies and transient/permanent immunomodulatory condition. Children with previous history of blood transfusion and therapy based on hyperimmune serum up to 90 days before peripheral blood collection were not recruited to this study. All tests were carried out in a blind fashion without knowing whether the samples were from the first or second arm.

### Testing Procedures

#### YF-Specific Neutralizing Antibodies

Heparin plasma samples were obtained from each volunteer and submitted to Ecteola-cellulose pre-treatment to remove heparin, as previously described by Campi-Azevedo et al. (19), for subsequent use in the PRNT assay. Ecteola-cellulose treated samples were assayed by the micro-PRNT_50_ test according to Simões et al. (20). For the present study, the performances (sensitivity, specificity, and global accuracy) of micro-PRNT_50_ and micro-PRNT_90_ were defined for children samples and the cut-off 1:10 in reciprocal of serum dilution and the micro-PRNT_50_ were selected as the most accurate condition. The micro-PRNT_50_ was performed at the Laboratório de Tecnologia Virológica, Bio-Manguinhos (LATEV, FIOCRUZ-RJ, Brazil). The results were expressed as reverse of sample dilution, considering seropositivity as PRNT titers higher than 1:10 sample dilution.

#### YF-Specific Phenotypic and Functional Memory

The analysis of 17DD-YF specific cellular memory response was carried out as previously described by Campi-Azevedo et al. (8). Briefly, *in vitro* 17DD-YF-specific peripheral blood lymphoproliferative assay were conducted in two separate batches, referred as: non-stimulated Control Culture and 17DD-YF Culture. After the long-term incubation (144 h), cultured cells were harvested and stained with Live/Dead Dye (Life Technologies, Carlsbad, CA, USA) and a mix of monoclonal antibodies (mAbs) to identify memory T-cell subpopulations (anti-CD4/RPA-T4/FITC; anti-CD8/SK1/PerCP-Cy5.5; anti-CD27/M-T271/PE, and anti-CD45RO/UCHL1/PE-Cy7) and memory B-cell subsets (anti-IgD/IA6-2/FITC, anti-CD27/M-T271/PE, and anti-CD19/HIB19/PerCP). In a parallel, an aliquot of cultured cells were incubated with Live/Dead Dye, labeled with anti-CD8/SK1/PerCP and after pre-fix/permeabilization procedure re-incubated with a cocktail of anti-cytokine mAbs (anti-TNF-α/MAb11/PE-Cy7; anti-IFN-γ/B27/Alexa-Fluor488, and anti-IL-5/JES1-39D10/PE). All monoclonal antibodies were purchased from BD Biosciences (San Jose, CA, USA). Stained cells were washed, fixed and the data was acquired (100,000 lymphocytes/test) on a BD LSRFortessa Flow Cytometer (BD Biosciences, San Diego, CA, USA). FlowJo software (version 9.3.2, TreeStar, San Diego, CA, USA) was used to establish distinct gating strategies to quantify the memory T and B-cells subpopulations as previously described (8), including: “T-cell memory subsets:” Naïve T-cells/(NCD4;NCD8)/CD27^+^CD45RO^−^; early Effector Memory T-cells/(eEfCD4;eEfCD8)/CD27^−^CD45RO^−^; Central Memory T-cells/(CMCD4;CMCD8)/CD27^+^CD45RO^+^ and Effector Memory T-cells/(EMCD4;EMCD8)/CD27^−^CD45RO^+^ and “B-cell memory subsets:” Naïve B-cells/(NCD19)/CD27^−^IgD^+^; Non-classical Memory B-cells/(nCMCD19)/CD27^+^IgD^+^ and Classical Memory B-cells/(CMCD19)/CD27^+^IgD^−^. The percentage of cytokine^+^ CD8^+^ T-cells was also quantified. The results were reported as 17DD-YF-stimulated Culture/non-stimulated Control Culture Index, calculated as the ratio of results observed in the 17DD-YF-stimulated Cultures divided by the respective non-stimulated Control Culture.

#### Data Analysis

The GraphPad Prism software, Version 5.0 (San Diego, CA, USA) was employed to perform all statistical analyses. Kolmogorov-Smirnov, D'Agostino and Pearson omnibus and Shapiro-Wilk normality tests were used to check data distribution. Multiple strategies were employed for data analysis. Kruskal-Wallis test followed by Dunn's post-test were employed for intergroup comparative analysis. The Chi-square test was used to compare seropositivity rates, biomarker signatures, descriptive analysis of selected biomarkers and analysis of resultant memory. Spearman's correlation test was carried out to determine the PRNT wane along time continuum. In all cases, significant differences were considered at *p* < 0.05.

Biomarker signature analysis was carried out using the 75th percentile (3rd quartile) values for each biomarker (17DD-YF-stimulated Culture/non-stimulated Control Culture Index) as the cut-off edge to identify subjects with high biomarker levels. Those biomarkers with more than 25% of subjects above the cut-off were considered for comparative analysis amongst groups. Venn diagram analysis (http://bioinformatics.psb.ugent.be/webtools/Venn/) was employed to identify biomarkers observed selectively in PV (days 30–45), referred as correlates of protection. Overlay of ascendant biomarker signatures were employed for comparative analysis of time-depended changes in immunological profile after primary vaccination.

Logistic and multinomial regression models were constructed to evaluate the association between time after vaccination and changes in the biomarker levels. Following this, the fitted regression model was employed to calculate the predicted probabilities for each biomarker (isolated or combined) along time continuum. The Receiver Operating-Characteristic curves (ROC) were was constructed to estimate the capacity of time as a predictor of changes in biomarkers levels to monitor the 17DD-YF memory after primary vaccination in children. The Area Under the ROC Curve (AUC) was used for comparative analysis of predictive capacity amongst biomarkers (isolated or combined).

## Results

### Progressive Time-Dependent Decrease in Neutralizing Antibody Titers Is Observed After 17DD-YF Primary Vaccination in Children

**Figure 1:**
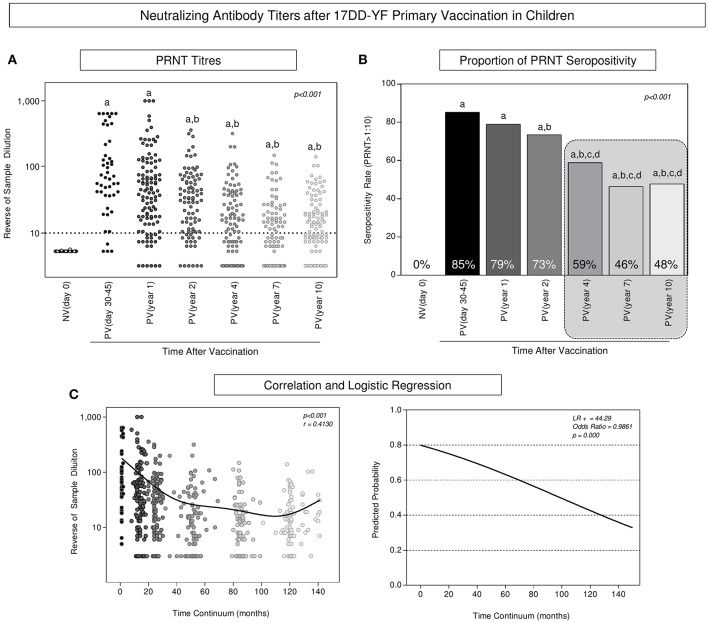
Neutralizing antibody titers after 17DD-YF primary vaccination in children. PRNT titers were measured in Ecteola-treated plasma samples (19) from non-vaccinated children at baseline NV(day 0)/(○, *n* = 47) and at different times after primary vaccination: PV(day 30–45)/(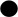, *n* = 47), PV(year 1)/(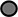, *n* = 141), PV(year 2)/(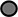, *n* = 114), PV(year 4)/(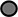, *n* = 128), PV(year 7)/(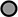, *n* = 116), and PV(year 10)/(°, *n* = 127), as described previously by Simões et al. (20). **(A)** The PRNT levels were expressed in reverse of serum dilution. **(B)** Proportion of PRNT seropositivity (PRNT>1:10) were calculated for each group and the results expressed as seropositivity rates at baseline NV(day 0) [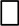] and at different times after primary vaccination: PV(day 30–45) [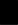], PV(year 1) [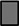], PV(year 2) [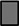], PV(year 4) [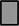], PV(year 7) [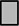], and PV(year 10) [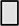], considering the serum dilution >1:10 as the cut-off (dashed line). **(C)** Correlation and logistic regression were employed to determine the wane of PRNT levels along time continuum and the results expressed as reverse of serum dilution and predicated probability, respectively. Statistical analysis was carried out as described in Methods. In all cases, significant differences at *p* < 0.05 were underscored by using letters “a,” “b,” “c,” and “d” for comparisons with NV(day 0), PV(day 30–45), PV(year 1), and PV(year 2), respectively and the *p*-values provide in the figure. Spearman correlation indices as well as Likelihood and Odds ratio are provided in the figure. Gray rectangle highlights the critical decrease of PRNT seropositivity rates ≥4 years after primary vaccination.

The levels of neutralizing antibodies, the proportion of PRNT seropositivity along with the correlation between PRNT levels and the logistic regression analysis of PRNT levels along time continuum are presented in the Figure 1. Data analysis revealed that primary vaccination induced a significant increase in the PRNT levels (Figure 1A) reaching a seropositivity rate of 85% in PV(day 30–45) as compared to NV(day 0) (Figure 1B). A progressive decrease in the PRNT levels was observed along time as early as 2 years after primary vaccination as compared to PV(days 30–45) (Figure 1A). Critical seropositivity rates (below 60%) were observed amongst vaccinees, particularly ≥4 years after primary vaccination (Figure 1B, gray dashed rectangle). Correlation analysis further supports the waning phenomenon observed in the PRNT levels along time after primary vaccination in children (Figure 1C). Furthermore, the outstanding likelihood ratio (LR^+^ = 44.29) and odds ratio (OR = 0.9861, 95% CI = 0.9819–0.9903) reinforce the abrupt and progressive decline month to month in PRNT levels along time continuum, which reached values of 0.8454, 0.6025, and 0.1865 at 12, 36, and 120 months, respectively (Figure 1C).

### Primary 17DD-YF Vaccination in Children Elicits Short-Lived YF-Specific Cellular Memory Mediated by Effector CD4^+^ and CD8^+^ T-Cells and Non-classical B-Cells

**Figure 2:**
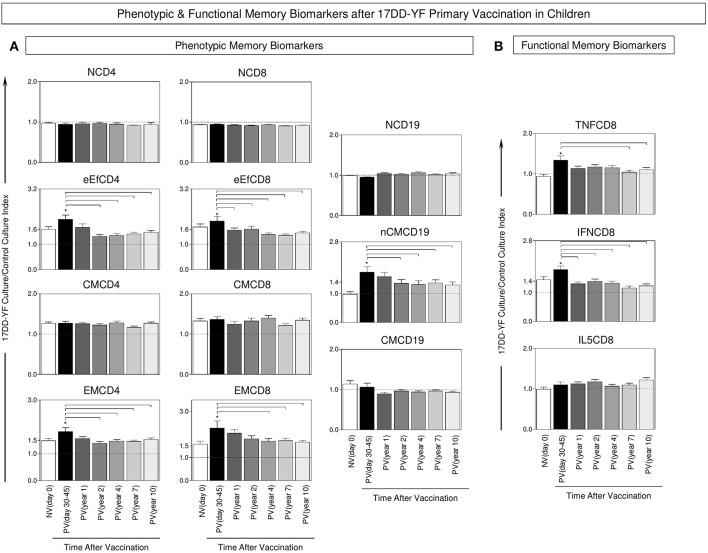
Phenotypic and functional memory biomarkers after 17DD-YF primary vaccination in children. The analysis of 17DD-YF-specific memory was measured upon *in vitro* 17DD-YF antigen recall as described previously by Campi-Azevedo et al. (8) for non-vaccinated children at baseline NV(day 0)/(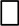, *n* = 47) and at different times after primary vaccination: PV(day 30–45)/(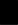, *n* = 47), PV(year 1)/(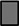, *n* = 141), PV(year 2)/(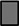, *n* = 114), PV(year 4)/(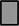, *n* = 128), PV(year 7)/(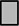, *n* = 116), and PV(year 10)/(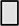, *n* = 127). **(A)** Flow cytometric staining were used to quantify phenotypic features of T-cell memory subsets: Naïve T-cells/(NCD4;NCD8)/CD27^+^CD45RO^−^; early Effector Memory T-cells/(eEfCD4;eEfCD8)/CD27^−^CD45RO^−^ Central Memory T-cells/(CMCD4;CMCD8)/CD27^+^CD45RO^+^ Effector Memory T-cells/(EMCD4;EMCD8)/CD27^−^CD45RO^+^ and B-cell memory subsets: Naïve B-cells/(NCD19)/CD27^−^IgD^+^; Non-classical Memory B-cells/(nCMCD19)/CD27^+^IgD^+^ and Classical Memory B-cells/(CMCD19)/CD27^+^IgD^−^. **(B)** Flow cytometric staining were also performed to quantity functional CD8^+^ T-cells producing TNF-α, IFN-γ and IL-5. The data were reported as median values ± inter-quartile range for 17DD-YF-stimulated Culture/non-stimulated Control Culture Index as described in Methods, highlighting the equivalence ratio by dashed line (Index = 1.0). Significant differences at *p* < 0.05 were underscored by using asterisk (*) to identify differences between NV(day 0) vs. PV(day 30–45) and intergroup differences identified by connecting lines.

The analysis of YF-specific phenotypic and functional biomarkers was evaluated upon *in vitro* 17DD-YF antigen recall and the results are presented in Figure 2. An increase of memory T-cells (eEfCD4, eEfCD8, EMCD4, and EMCD8) as well as nCMCD19 cells along with an up-regulation of TNF-α and IFN-γ produced by CD8^+^ T-cells is observed in PV(day 30–45) as compared to NV(day 0) (Figures 2A,B). Moreover, a significant decrease in these biomarkers occurs along the time, particularly ≥4 years after primary vaccination when almost all these attributes decline as compared to PV(days 30–45) (Figure 2).

### Biomarker Signatures Emphasize the Short-Term Persistence of Phenotypic Effector Memory and Functional Activity of CD8^+^ T-Cells

**Figure 3:**
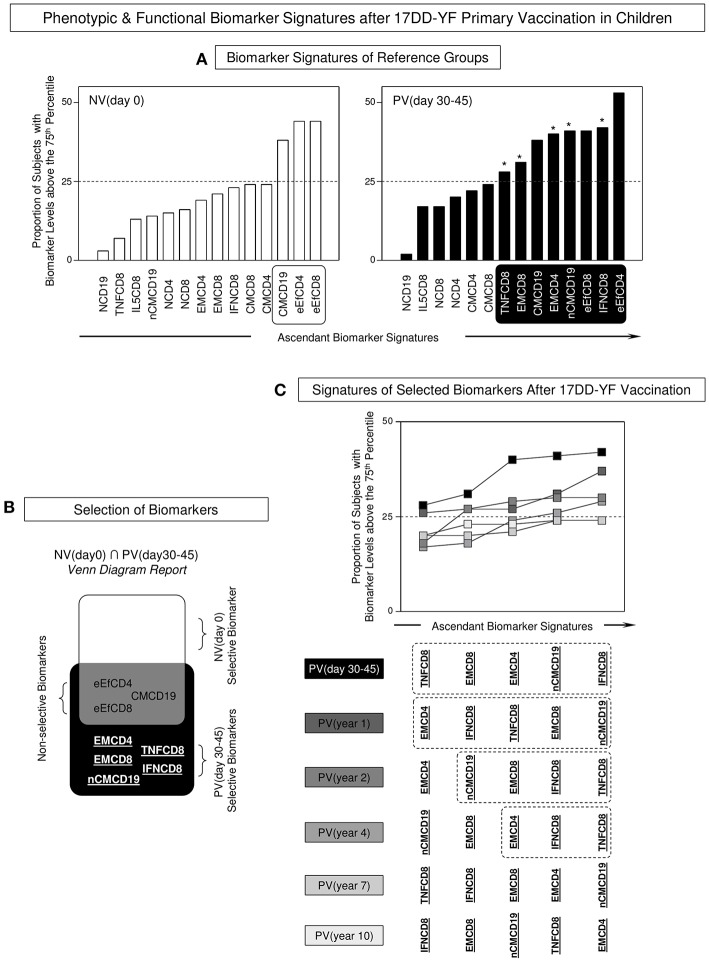
Phenotypic and functional biomarker signatures after 17DD-YF primary vaccination in children. **(A)** Biomarker signatures of reference groups NV(day 0) [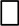] and PV(day 30–45) [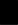] were assembled to select biomarkers above the 75th percentile with proportions higher than the 25% in each group (white/black background rectangles). The selected biomarkers were underscored by asterisk (*) to identify differences between NV(day 0) vs. PV(day 30–45). **(B)** Venn diagram report was employed to identify the set of biomarkers selectively increased in [PV(day 30–45) vs. NV(day 0)]. The attributes EMCD4, EMCD8, nCMCD19, TNFCD8, and IFNCD8 were underscored as PV (day 30–45)-selective biomarkers. These attributes were tagged in bold underline format and employed for follow-up analysis overtime after 17DD-YF primary vaccination. Biomarkers with proportion higher than the 25% were underscored by asterisk (*) to identify differences between NV(day 0) vs. PV(day 30–45). **(C)** Overlaid signatures of selected biomarkers were assembled to identify changes in the 17DD-YF specific phenotypic and functional features at different times after primary vaccination: PV(day 30–45) [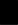], PV(year 1) [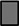], PV(year 2) [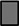], PV(year 4) [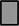], PV(year 7) [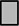], and PV(year 10) [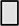]. Dashed rectangles underscore the critical decline of selected biomarkers overtime after primary vaccination with absence of EMCD8 ≥ 4 years after primary vaccination.

In order to accomplish the characterizing of the duration of phenotypic and functional memory induced by the 17DD-YF primary vaccination in children, the biomarker signatures were built for comparative analyses along time. To accomplish this goal, initially overlaid biomarkers signatures of NV(day 0) vs. PV(days 30–45) were assembled to identify attributes selectively observed in PV(day 30–45), further referred as correlates of protection in children (Figure 3A). Venn diagram analysis indicated that besides three common attributes (eEfCD4, eEfCD8, and CMCD19), five biomarkers (EMCD4, EMCD8, nCMCD19, TNFCD8, and IFNCD8) were tagged to be employed as selective correlates of protection for follow-up analysis overtime after primary 17DD-YF vaccination (Figure 3B).

Once the correlates of protection for follow-up analysis were selected, overlaid biomarker signatures were constructed to verify changes along time upon 17DD-YF primary vaccination (Figure 3C). Data analysis pointed out that all five biomarkers were persistently observed in PV(day 30–45) and PV(year 1). Although some attributes were not observed in PV(year 2), the lack of EMCD8, considered one of the top biomarkers to monitor the immunological memory to 17DD-YF vaccine (9) was noticed in PV(year 4), PV(year 7), and PV(year 10) (Figure 3C).

### Descriptive Analysis Confirms the Time-Dependent Decrease of Proxies of Protection Upon 17DD-YF Primary Vaccination in Children

**Figure 4:**
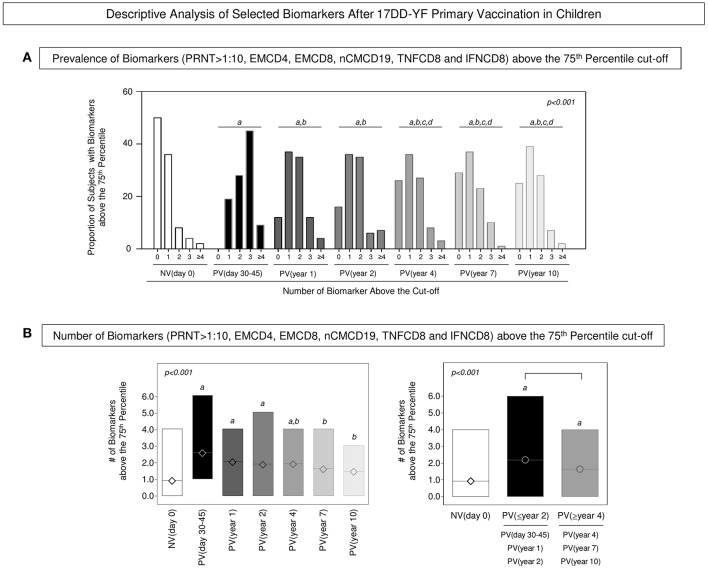
Descriptive analysis of selected biomarkers after 17DD-YF primary vaccination in children. **(A)** Prevalence of Biomarkers (PRNT>1:10, EMCD4, EMCD8, nCMCD19, TNFCD8, and IFNCD8) above the 75th percentile cut-off. Data are presented as proportion of subjects with biomarkers above the cut-off at baseline NV(day 0) [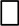] and at different times after primary vaccination: PV(day 30–45) [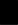], PV(year 1) [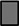], PV(year 2) [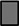], PV(year 4) [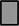], PV(year 7) [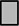], and PV(year 10) [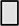]. **(B)** Number of Biomarkers (PRNT>1:10, EMCD4, EMCD8, nCMCD19, TNFCD8, and IFNCD8) above the 75th Quartile cut-off. Data are presented as mean (min to max) number of biomarkers above the cut-off at baseline NV(day 0) [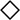] and at different times after primary vaccination: PV(day 30–45) [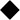], PV(year 1) [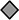], PV(year 2) [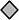], PV(year 4) [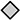], PV(year 7) [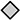], and PV(year 10) [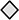] as well as PV(≤year 2) [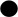], PV(≥year 4) [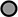]. Significant differences at *p* < 0.05 are underscored by using letters “a,” “b,” “c,” and “d” for comparisons with NV(day 0), PV(day 30–45), PV(year 1), and PV(year 2), respectively. Intergroup differences are identified by connecting lines. The *p*-values are provided in the figure.

The biomarkers tagged as correlates of protection (EMCD4, EMCD8, nCMCD19, TNFCD8, and IFNCD8) were employed together with the neutralizing antibody levels to carry out a descriptive analysis to monitor the YF-specific memory along time after 17DD-YF primary vaccination. For this purpose, the proportion of subjects displaying biomarkers levels above the 75th percentile cut-off and PRNT levels >1:10 were calculated and data reported for each group (Figure 4A). Data demonstrated that in NV(day 0) there is a predominance of subjects displaying 0–1 biomarkers above that cut-off. Conversely, in the PV(day 30–45) there is a significantly higher prevalence of subjects displaying 3 biomarkers above that cut-off. In PV(year 1) and PV(year 2) there was a balanced proportion of subjects displaying 1–2 biomarkers above that cut-off. Notably, in PV(year 4), PV(year 7), and PV(year 10) there was a higher prevalence confined in only one biomarker above that cut-off (Figure 4A). Complementary analysis further revealed that the median number of biomarkers above that cut-off found in PV(day 30–45), PV(year 1), and PV(year 2) was higher as compared to NV(day 0). Although in PV(year 4), the median value still differed from that observed in NV(day 0), it was lower as compared to PV(days 30–45). Additionally, a critical median number of biomarkers above that cut-off was observed in PV(year 7) and PV(year 10) as compared to PV(days 30–45) that did not differ from those found in NV(day 0) (Figure 4B). Based on these findings, the vaccinees were distributed into two major groups referred as PV(≤ year 2) and PV(≥year 4). The results showed that both groups presented higher median number of biomarkers above the cut-off as compared to NV(day 0), although the PV(≥year 4) group exhibited lower values as compared to PV(≤ year 2) (Figure 4B).

### Predicted Probability Analysis Emphasizes That Neutralizing Antibody Levels (PRNT), EMCD8, and nCMCD19 Are the Top Biomarkers to Monitor the 17DD-YF Memory After Primary Vaccination in Children

**Figure 5:**
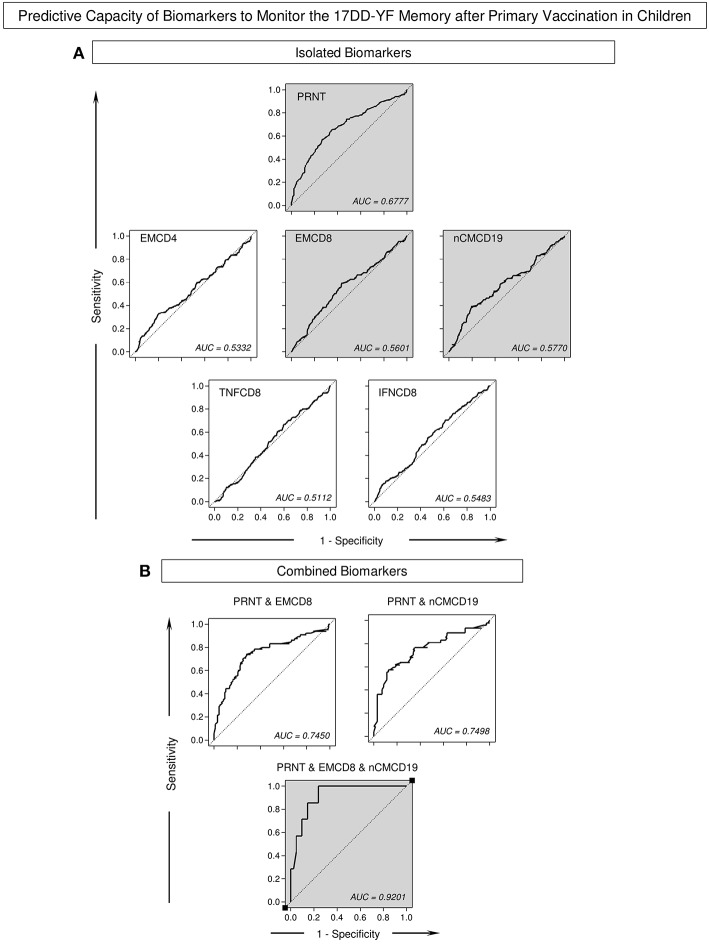
Predictive capacity of biomarkers to monitor the 17DD-YF memory after primary vaccination in children. The Receiver Operating Characteristic (ROC) curves were used to estimate the capacity of time as a predictor of changes in biomarker levels to monitor the 17DD-YF memory after primary vaccination in children. Logistic and multinomial regression models were constructed to evaluate the association between time after vaccination and changes in the biomarker levels. Following, the fitted regression model was employed to calculate the predicted probabilities for each biomarker **(A)** isolated or **(B)** combined along time continuum. The Area Under the ROC Curves (AUC) were employed for comparative analysis of predictive capacity amongst biomarkers and the values provided in the figure. The gray background highlights the top three isolated biomarkers and the best combination of predictor biomarkers to monitor the 17DD-YF memory after primary vaccination in children.

Logistic and multinomial regression models were constructed and the fitted regression model employed to calculate the predicted probabilities for each biomarker previously tagged as correlates of protection (EMCD4, EMCD8, nCMCD19, TNFCD8, and IFNCD8) to monitor the 17DD-YF memory after primary vaccination along time continuum. The ROC curves were constructed for comparative analysis of predicted capacity amongst biomarkers (isolated or combined). Based on the global accuracy (Area Under the ROC Curve—AUC), the neutralizing antibody levels (PRNT), EMCD8, and nCMCD19 presented moderate performance when employed isolated as a single parameter to monitor the 17DD-YF memory after primary vaccination in children (AUC = 0.6777; 0.5601, and 0.5770, respectively) (Figure 5A). The combined analysis of neutralizing antibody levels (PRNT), and EMCD8 and nCMCD19 further improved the predictive capacity of using these biomarkers to monitor the 17DD-YF memory after primary vaccination in children (AUC = 0.9201) (Figure 5B).

### The Resultant Memory (PRNT, EMCD8, or NCMCD19) Wanes Overtime Reaching Critical Values at 4 or More Years After 17DD-YF Primary Vaccination in Children

**Figure 6:**
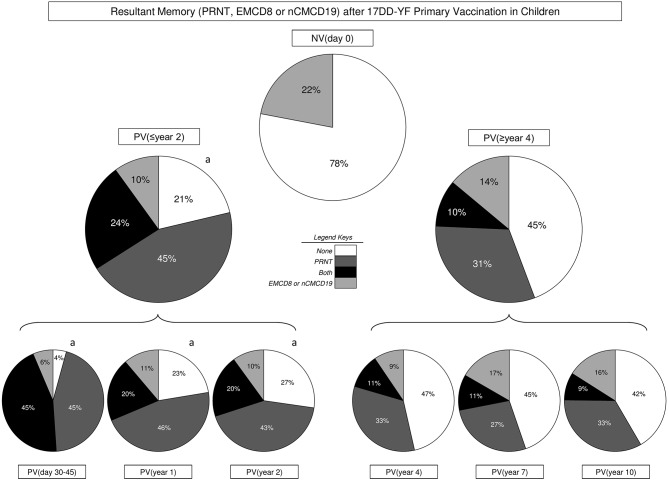
Resultant memory (PRNT, EMCD8, or nCMCD19) after 17DD-YF Primary vaccination in children. The resultant memory status (PRNT, EMCD8, or nCMCD19) were assessed at individual level to define the overall proportion of subjects presenting None (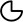), PRNT (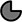), cellular memory “EMCD8 and/or nCMCD19” (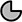) or both attributes “PRNT and cellular memory” (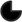) at distinct time-points before/after primary 17DD-YF vaccination, including: NV(day 0), PV(≤year 2), PV(≥year 4) as well as PV(day 30–45), PV(year 1), PV(year 2), PV(year 4), PV(year 7), and PV(year 10). Significant differences (*p* < 0.05) of resultant memory status amongst study groups were assessed by Chi-square test and represented by letter “a” as compared to NV(day 0).

The results of neutralizing antibody levels (PRNT), EMCD8, and nCMCD19 profiles were combined at individual level to build a memory matrix and calculate the resultant YF-specific memory, comprising of humoral (PRNT) and cell-mediated (EMCD8 or nCMCD19) immunity. Then, each volunteer was classified as they present “None,” “PRNT,” “EMCD8 and/or nCMCD19,” or “Both” attributes above the cut-off threshold, i.e., PRNT positivity at serum dilution >1:10, EMCD8 (17DD-YF-stimulated Culture/non-stimulated Control Culture >2.19) or nCMCD19 (17DD-YF Culture/Control Culture >1.66). The results demonstrated that 17DD-YF primary vaccination was able to guarantee persistent resultant memory in 79% of children included into PV(≤year 2). Conversely, a clear decline in the resultant memory down to 55% was observed into PV(≥year 4). Specifically, the resultant memory initially observed in 96% of children in PV(day 30–45) decrease to 77% in PV(year 1) and 73% in PV(year 2) followed by a marked shift down to 53, 55, and 58% in PV(year 4), PV(year 7) and PV(year 10), respectively (Figure 6).

## Discussion

The YF vaccination is currently recommended as a single dose for residents of disease risk areas and people traveling from or to those areas, who aged 9 months or older (5, 6, 21–23). According to the WHO, very few primary vaccine failures following YF vaccination have been reported. It has been proposed that, in addition to neutralizing antibodies, both innate and cell-mediated immunity also contribute to the initial immune response. Defining the parameters that modulate vaccine responses is relevant to increase vaccine effectiveness. It has been proposed that several factors may affect the YF vaccine response including: genetic background, gender, age, and environmental differences. Muyanja et al. (24) have proposed that host-specific immune response microenvironment may contribute to the effectiveness of the 17D-YF vaccine. These authors have suggested that an activated immune microenvironment prior to vaccination impedes the efficacy of the 17D-YF vaccine in an African cohort and suggest that booster regimens should be proposed to improve efficient immunity after YF vaccination. Other studies have suggested that the ability of YF-17D vaccine to infect dendritic cells and activate multiple Toll-like receptors seems to be essential for generating a potent immune response after vaccination (17, 25). A distinct hypothesis have been tested to explain the lower seropositivity rates after YF vaccination in children. An observational multicenter study, carried out by the Collaborative Group for Studies on Yellow Fever Vaccines has reported that the 17DD-YF vaccine reached distinct seroconversion rates in children according to the age at vaccination (26). Moreover, it has been demonstrated that simultaneous administration of other viral vaccines reduces significantly the response to YF vaccine in children (15). Conversely, no association between seroconversion rates and the maternal immunity status to YF has been observed (27). Our group have previously observed that children non-responsive to primary 17DD-YF vaccine presented a striking lack of innate immunity pro-inflammatory response, specially low levels of IL-12^+^ and TNF-α^+^ neutrophils and monocytes, along with an increased regulatory profile in the adaptive response, including higher levels of IL-4^+^CD4^+^ T cells as well as IL-10^+^ and IL-5^+^CD8^+^ T cells (28). Interestingly, the revaccination of children with primary vaccination failure was able to restore the innate and adaptive immunity toward a balanced pro-inflammatory/regulatory profile.

Some studies that investigated the impact of booster doses on the status of YF-specific immune response in adults postulated that booster vaccination did not increase the titers of YF-specific antibodies nor induced or altered the phenotypes of CD8^+^ T-cells and the immune responses observed following revaccination were reduced compared to primary responses (23, 29, 30). However, other studies have demonstrated that booster doses are relevant to guarantee the long-term persistence of 17DD-YF-specific memory components in travelers (31) and in residents of areas with YF-virus circulation (32, 33). Therefore, the single dose recommendations for YF vaccines have been considered controversial.

The decision to no longer recommend booster vaccination may especially have a direct impact on children, given that besides higher primary vaccination failures (15), there is no evidence for the long-term persistence of protective immunity in primary vaccinated children. Our group has already described the occurrence of time-dependent loss of YF-immunity in primary vaccinated adults (7–9). If this scenario also occurs in children it would contribute to worsening the setting in which a large proportion of individuals would become exposed to potential risk of YF infection, especially in endemic areas of high virus circulation. As indicated by the results of the present study, a substantial proportion of children could be susceptible to YF-virus infection, especially in endemic areas.

The studies that evaluated the timeline kinetics of correlates of protection after 17D-YF and 17DD-YF vaccination in adults have demonstrated that both humoral and cellular-mediated YF-specific immunity display a relevant decline overtime in single dose recipients of YF vaccines and that the booster doses efficiently improve the immunological status in re-vaccinated recipients (29–33). Recent studies from our group have shown that secondary or multiple vaccination regimens in adults are able to further improve the immunity parameters triggered by primary 17DD-YF vaccination and restore the resultant YF-specific memory in 100% of the volunteers. Moreover, it was observed that all vaccinees had at least one or both proxy of protection detectable at ≥10 years post-secondary vaccination (33).

Previous studies from our group have compared the cytokine-mediated immune response triggered by 17D-213/77-YF or 17DD-YF vaccines in children submitted to primary vaccination at 9–12 month of age with those non-responders to primary vaccination and also with those that received (28, 34). The results demonstrated that all children that received a booster dose 1 year after primary vaccination failure seroconverted and shifted the overall cytokine signatures toward a balanced pro-inflammatory/regulatory response of innate and adaptive immunity, overcoming the striking lack of innate immunity pro-inflammatory response observed in non-responder children (28, 34). These studies clearly indicated that booster regimens are relevant to guarantee the persistence of long-term immunity in areas with high risk of yellow fever transmission.

Data about the duration of YF-specific immunity in children following primary vaccination is still scarce and can provide supporting evidence to support the public health programs for YF control worldwide. In this sense, the present investigation was designed to explore the duration of humoral and cellular immunity in primary vaccinated children in a 10-years cross-sectional timeline.

Our findings indicate that a substantial proportion of children lose their antiviral humoral immunity at 4 or more years after 17DD-YF primary vaccination. The PRNT has been considered the classical gold standard to measure post-vaccination immunity to YF for decades and generally regarded as the most appropriate parameter for monitoring protection by YF vaccine (6, 16). Reports from Niedrig et al. (16) regarding the timeline kinetics of YF-neutralizing antibody after 17D-YF vaccination in adults demonstrated that seropositivity rates (>1:10) significantly decrease from 94.0 to 74.5% from 1 to 10 years, respectively. In fact, upon closer inspection, the data clearly showed that antibody titers declined more rapidly during the first 1–4 years after 17D-YF vaccination and that seropositivity rates (>1:10) reached 69% when vaccinees are grouped together from 5 to 35 years post vaccination (13, 16). This rapid decline in neutralizing antibodies early after YF vaccination was also in a study by Hepburn et al. (29), which demonstrated that antibody levels decayed within 3–4 years in approximately half of the adult vaccines, even after booster vaccination.

Short-lived persistence of cell-mediated immunity has also been observed in memory T and B-cells (EMCD4; EMCD8; TNFCD8; IFNCD8; nCMCD19) after 4-years after a 17DD-YF single dose. Complementary predicted probability analysis together with resultant memory assessment highlighted that besides neutralizing antibody levels (PRNT), the levels of EMCD8 and nCMCD19 also decline to critical values at ≥4-years after primary vaccination. Several studies strongly suggested that CD8^+^T cells are relevant for immune protection upon YF after primary or secondary vaccination (8, 9, 17, 35). The role of nCMCD19 cells has not been completely elucidated in YF-vaccinated recipients. It is known that the maintenance of long-lived plasma cells that secrete antigen-specific antibodies, as well as memory B-cells, is essential for protection against pathogens, and is the basis of successful vaccinations (36). The nCMCD19 cells (IgD^+^CD27^+^CD19^+^) are known as unclass-switched cells with similar functions compared to classical switched memory B-cells (IgD^−^CD27^+^CD19^+^) and are not in the process of transition from naive to memory B cells. These nCMCD19 cells are believed to play an important role in secondary immune response in early phases of infection (37).

The current study further indicates that booster vaccination regimen may be required to guarantee protective immunity in children. The rapid and expressive loss of humoral and cellular immunity in a subpopulation of primary vaccinated children suggests that the first booster dose of vaccine should be administered within 4–5 years after primary vaccination instead of 10 years after vaccination as proposed previously for adults (32, 33).

The study's limitations include the cross-sectional representation of the average immune status of several birth cohorts, which assumed they differ only in time after vaccination, although this seems a legitimate assumption given that the vaccine and immunization practices have remained unchanged in the time period of those cohorts. Another limitation could be the possibility of booster by natural infections, as those children lived in an area where YF vaccine is recommended. However, it is important to mention that the study participants lived in a metropolitan region where there had been no cases in humans and non human primates.

The inclusion of the YF vaccine into worldwide immunization program for children living in endemic areas represents an important measure to ensure the YF-immunization in infancy and guarantee the effective control of YF expansion. The knowledge about duration of correlates of protection following YF vaccination in children is relevant to support further decisions to be made regarding the need of revaccination and to define the precise time for booster regimens. The results presented here add scientific knowledge about the immune response induced by 17DD-YF vaccine and bring new insights and increase awareness for healthcare workers about the importance of YF vaccination/revaccination in childhood. Moreover, our results may support the revision of the recommendation for a single dose YF-vaccine and will certainly guarantee that a large contingent of children will be revaccinated, improving the prevention of YF. Altogether, these data would be helpful to define targets and indicators for protection and susceptibility, especially in endemic countries with high historical rates of YF virus circulation in continuous expansion.

## Data Availability

All datasets generated for this study are included in the manuscript/supplementary files.

## Ethics Statement

This studies involving human participants were reviewed and approved by the research ethics committee at the Escola Nacional de Saúde Pública (CAAE 0014.0.031.000-10, February 20th 2010) as well as at Instituto René Rachou (CAAE 0023.0.245.000-10, February 11th 2011 and CAAE 25315213.6.0000.5091, May 23rd 2015) and registered at the Clinicaltrials.gov (NCT 02990182, January 9th 2015). Written informed consent to participate in this study was provided by the participants' legal guardian/next of kin.

## Author Contributions

AC-A, IC, LC, MM, MF, RM, AH, PT, PV, AR, CD, AT-C, and OM-F: designing research study. IC, LC, AR, CD, and OM-F: funding acquisition. AC-A, LR, VP-M, JC, LA, CF, CC-P, ES-F, TN, SL, and MS: conducting experiments. JL, JR, and LC: field study. AC-A, LA, SL, and MS: acquiring data. AC-A, LR, VP-M, JC, LA, CF, CC-P, IC-R, and JM: analyzing data. PT and PV: validation. AH, PT, and PV: advisory committee. AC-A, LR, JC, CC-P, IC-R, AT-C, and OM-F: writing the manuscript.

### Conflict of Interest Statement

The authors declare that the research was conducted in the absence of any commercial or financial relationships that could be construed as a potential conflict of interest.
